# Blood Lead Levels and Risk Factors for Lead Exposure in a Pediatric Population in Ho Chi Minh City, Vietnam

**DOI:** 10.3390/ijerph15010093

**Published:** 2018-01-08

**Authors:** Deborah Havens, Minh Hong Pham, Catherine J. Karr, William E. Daniell

**Affiliations:** 1 Department of Environmental and Occupational Health Sciences, University of Washington, Seattle, WA 98195, USA; ckarr@uw.edu (C.J.K.); bdaniell@uw.edu (W.E.D.); 2 Liverpool School of Tropical Medicine, Liverpool L3 5QA, UK; 3 Children’s Hospital #2, University of Medicine and Pharmacy, Ho Chi Minh City, Vietnam; minhhong1964@yahoo.com.vn

**Keywords:** lead, child, environmental exposure, Vietnam

## Abstract

Although lead recycling activities are a known risk factor for elevated blood levels in South East Asia, little is known regarding the prevalence of and risk factors for elevated blood lead levels (BLL) among the general pediatric population in Vietnam. This study is a cross-sectional evaluation of 311 children from Children’s Hospital #2 in Ho Chi Minh City, Vietnam. Capillary blood lead testing was performed using the LeadCare II. Mean BLLs were 4.97 μg/dL (Standard Deviation (SD) 5.50), with 7% of the participants having levels greater than 10 μg/dL. Living in Bing Duong province (OR 2.7, 95% CI 1.4–5.6.1) or the Dong Nai province (OR 2.3, 95% CI 1.0–5.1) and having an age greater than 12 months (OR 6.0, 95% CI 3.1–11.8) were associated with higher BLLs. The prevalence of elevated BLLs in Vietnam is consistent with other SE Asian countries. Mean BLLs in Ho Chi Minh City are markedly less than those seen in a separate study of children living near lead recycling activities. Additional evaluation is necessary to better detail potential risk factors if screening is to be implemented within Vietnam.

## 1. Introduction

Childhood lead poisoning is one of the most extensively studied and documented environmental exposures worldwide. However, despite awareness that there is no known safe level of lead exposure [1] and that lead poisoning is preventable, WHO estimated that lead was responsible for 13.9 million disability associated life years (DALYs) in 2012 and contributed to mild to moderate mental retardation in 0.6 million children annually [2,3]. In 2015, the Global Burden of Disease study attributed 9.3 million DALYs to lead exposure, with 0.5 million premature deaths, 2.5% of the global burden of heart disease [4] and 12.4% of the global burden of idiopathic intellectual disability [5].

Sources of environmental lead tend to differ markedly between nations. In developed countries, such as the United States (US), children most at risk for lead exposure are often exposed through peeling paint in dilapidated housing or due to leaded pipe solder. In developing countries, the exposures are commonly associated with occupational activities such as smelting, mining, or recycling [6]. Additionally, lead may be added to paints, pigments, toys, traditional medicines, and cosmetics—exposures that do not remain confined to their country of origin.

The greatest exposure concern for the general population, leaded gasoline, has been removed from most countries worldwide and subsequent blood lead levels have declined steadily [7,8,9,10]. In the 1970s, when US federal legislation was enacted to eliminate the use of leaded gasoline, the median blood lead level (BLL) of US children ages 1–5 was 15 µg/dL (from 1976 to 1980). In 1988–1991, the median was 3.6 µg/dL, and by 1999 the median BLL had decreased to 1.9 µg/dL [11]. Leaded gasoline was phased out in Vietnam in 2001 [12]. Unfortunately, no surveillance BLL data is available to confirm that Vietnamese children are now at decreased risk of complications due to lead exposure. Even with the elimination of leaded fuel, other developing countries have seen elevated BLLs due to environmental lead exposures such as those due to lead battery recycling and mining [13,14]. Some of these same exposures have been documented throughout Vietnam [15].

There is a great deal of variation between countries in their approach to pediatric lead evaluation. The US has a comprehensive approach with screening criteria to target children who are most likely at risk [11]. However, at the time of this project, Vietnam did not have any routine surveillance in place, although there has been increasing awareness of at-risk children, particularly around lead recycling and craft villages. Notably, elevated lead levels in children have been seen in Dong Mai, a village near Hanoi wherein lead battery recycling occurs [16]. One goal of this study was to increase awareness and understanding of pediatric lead exposures in Vietnam, utilizing a cross-sectional analysis to determine the prevalence of increased BLL and risk factors for lead exposure in a general population of Vietnamese children. This study was also intended to provide a useful reference comparison for the ongoing evaluations of children in villages containing lead battery recycling establishments.

## 2. Materials and Methods

This study, a collaboration between the University of Washington in Seattle, Washington, USA and personnel at Children’s Hospital #2 in Ho Chi Minh City (HCMC), Vietnam, evaluated a cross-sectional sample of children in Southeastern Vietnam, by measuring BLLs and assessing possible lead exposure predictors through the completion of a questionnaire by the parents. All participants were recruited from the Respiratory Ward of Children’s Hospital #2, during April and May 2012. The study was approved by the Ethics Committee at Children’s Hospital #2 and by the Institution Review Board (IRB) Human Subjects Division at the University of Washington (UW #42486).

### 2.1. Study Population and Sample Selection

Children’s Hospital #2 is the second largest pediatric hospital in HCMC. It serves HCMC and the surrounding provinces in Southeastern Vietnam. Children hospitalized during April–May 2012 in the Respiratory Ward, aged 0–15 years, whose parents or guardians provided informed and written consent, were potentially eligible to participate, and all who were eligible agreed to take part in the study. To meet the IRB requirement to minimize procedures on hospitalized children, the children were only eligible if they would require blood draws during their hospital stay. The Vietnamese resident physicians involved in the research project confirmed written adult consent and child assent forms. The children and their guardians were not provided any remuneration for their participation. The child’s blood sample was tested for lead using the LeadCare II Blood Lead Test System (Magellan Diagnostics, Inc., North Billerica, MA, USA). All parents and guardians were provided with educational materials regarding lead exposure. If the participant had a hemoglobin (HgB) and/or hematocrit (Hct) drawn during their hospitalization, those lab results were collected. Heights and weights of the children were also obtained. 

### 2.2. Questionnaire

Demographic information about the participants, including age, sex, schooling, and clinical symptoms was obtained via questionnaire. Information was also collected about parental occupations and educational levels, potential lead recycling in the household or neighborhood, use of lead battery casings in or outside of the home, use of lead cookware, household sources of water, hobbies or craft work entailing potential lead exposure, and whether any individuals in the household had ever had a blood lead test.

### 2.3. Blood Sampling and Analysis

Prior to drawing blood for the child’s daily labs, his or her skin was wiped for approximately 30 s with a LeadTech™ (MEDTOX Scientific, St. Paul, MN, USA) wipe to remove any possible lead surface contamination. The skin was then disinfected with the usual methods used by the nursing staff (alcohol swab or cotton ball soaked with alcohol). Blood (50 µL) was collected in a heparinized capillary tube (provided by the LeadCare II test kit) via fingerstick. The use of capillary fingerstick was a routine procedure for acquiring blood from infants and children at this hospital. Capillary-tube blood was transferred to a LeadCare reagent tube, mixed thoroughly, and then immediately refrigerated until analysis. Analysis was completed within 48 h with a LeadCare II analyzer.

The LeadCare II is a point of care Clinical Laboratory Improvement Amendment (CLIA) certified device that utilizes electrochemistry (anodic stripping voltammetry) to detect lead. Red blood cells from the sample lyse when mixed with a dilute hydrochloric acid reagent, releasing the lead. The analyzer applies electrical potential that causes lead to collect on the sensor, which can then be measured [17]. In comparison with samples tested using Graphite Furnace Atomic Absorption Spectroscopy (GFAAS), the LeadCare II manufacturer noted a correlation *r*^2^ of 0.992, and the accuracy relative to GFAAS testing had a bias of +0.07 µg/dL for BLLs 0–10 µg/dL, +4.7% for BLLs 10.1–25.0 µg/dL and +5.0% for BLLs 25.1–65 µg/dL [18]. This device has been used for screening in many countries including the US and Australia. Prior to each day’s testing, the analyzer was calibrated with the supplied calibration equipment. For quality control, with each new test lot kit (48 samples), the analyzer was tested using the supplied control samples. The levels were within the acceptable range with each lot. In the product literature, the LeadCare II analyzer is expected to work well at 54–97 °F (12–36 °C) and at 12–80% relative humidity. Due to concerns about instability of the test kit and reagents in the warm and humid environment of HCMC, the LeadCare II analyzer was stored and used in the Children’s Hospital #2 Neonatal Intensive Care Unit, an air-conditioned ward kept at approximately 21 °C, with humidity under the recommended 80%.

### 2.4. Reporting

The LeadCare II has a reporting range of 3.3–65.0 µg/dL. Oral and written results of the blood lead testing were provided to the family on the day after the testing occurred or via telephone if the child had left the hospital. If a child’s level was found to be elevated, the family was interviewed regarding possible sources of lead exposure and recommendations were provided as appropriate. All the families were given education regarding lead avoidance and ways to decrease potential lead exposure. Additional testing was recommended for any children with levels greater than 10 µg/dL. Confirmatory venipuncture testing was conducted for any children (*n* = 3) with levels above 35 µg/dL, by the Experimental Analysis Center (EAC) in HCMC.

### 2.5. Data Analysis

Data was analyzed using SPSS (Version 19, IBM Analytics, Armonk, NY, USA). BLLs were log-transformed for regression analysis due to non-normal distribution. BLLs described as “low” were assigned a value of 1.65 (limit of detection/2) [19]. No “high” values were identified (>65 µg/dL). Logistic regression was used to examine the association between individual independent variables and dichotomized BLL (<5 µg/dL vs. ≥5 µg/dL). Variables that were found by initial analysis to be associated with elevated BLLs were further considered by sequential entry into multivariate logistic regression models. Logistic regression results are presented for dichotomization at 5 μg/dL due to the low prevalence of risk factors of concern and small number of children with elevated BLL. Numeric variables that had a normal distribution such as age, HgB, and Hct were analyzed using independent sample *t*-tests.

## 3. Results

### 3.1. Overall Distribution of BLL

**Figure 1:**
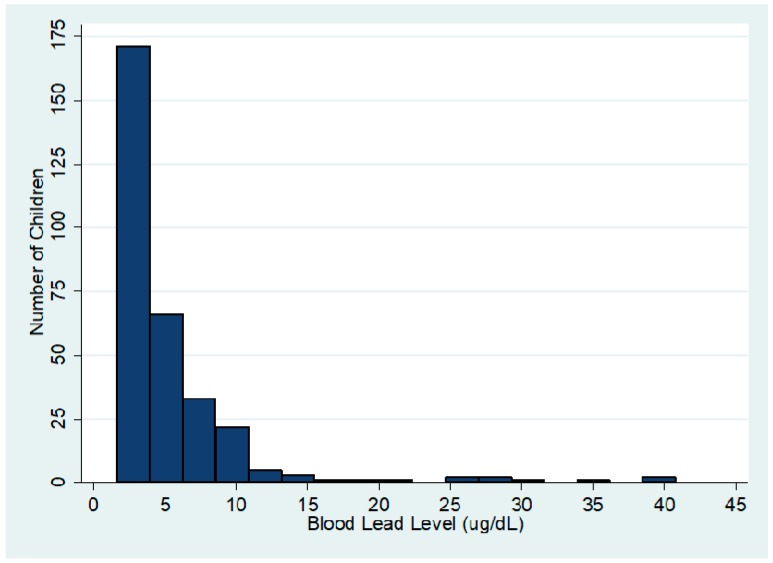
Distribution of blood lead levels (BLLs) (*n* = 311).

The distribution of BLLs is shown in Figure 1. Of the 311 children tested, 289 children (92.9%) had BLLs <10 µg/dL. Three children had levels >35 µg/dL with the highest measuring 40.8 μg/dL. The overall mean lead level in this population was 4.97 μg/dL (standard deviation, SD 5.50). Median level was 3.60 μg/dL (interquartile range, IQR 1.65–6.10).

### 3.2. Subject Characteristics

Table 1 displays characteristics of the children enrolled in the study, distinguishing between children who reside in HCMC and children from all other provinces. About half of the children came from HCMC (*n* = 145, 47%). There were more male (61%) than female (39%) children in the sample. Although children from HCMC and the other provinces showed a similar frequency for most variables, more potential lead exposures were reported outside of HCMC and water sources varied markedly between HCMC and the other provinces. Piped water was more commonly used in HCMC (45%) than elsewhere (22%), whereas well water use was reported more frequently in the other provinces (47%) than in HCMC (18%).

### 3.3. Geographic Distribution of BLL

There was a nearly two-fold difference across the range of median BLL values observed for children from each of the provinces represented in the study sample, and these differences were statistically significant (Table 2; *p* = 0.009, Kruskal–Wallis). The distribution of BLLs was alsosignificantly different when comparing children from HCMC with children from all other locations combined (HCMC 1.65 µg/dL, IQR 1.65–5.35; non-HCMC 3.8 µg/dL, IQR 1.65–6.70; *p* = 0.02, Kruskal–Wallis).

### 3.4. Child and Family Characteristics and Distribution of Blood Lead Levels

In general, mean BLLs were significantly lower in the youngest children (0–12 months old) (3.75 μg/dL; SD 5.61) compared to all older children (6.19 μg/dL; SD 5.13) (*p* < 0.001) (Table 3). Mean BLLs were highest in children age 1–2 years at 6.71 μg/dL (SD 6.63), compared to those age 0–1 years, age 2–3 years (5.39 μg/dL; SD 3.07) and for those greater than 3 years of age (5.70 μg/dL; SD 2.81). Mean BLLs were also higher among children who attended school outside the home (6.54 μg/dL (SD 5.21) vs. 4.46 μg/dL (SD 5.51) (*p* < 0.001)). There were no significant differences in BLLs relative to gender or any of the other environmental or occupational variables.

### 3.5. Risk Factors for High Blood Lead Levels

#### 3.5.1. Bivariate Analysis

Odds of having higher BLLs were associated with increased age (13–24 months: OR 4.4, 95% CI 2.4–8.1, *p* < 0.001; 25–36 months: OR 4.0, 95% CI 1.9–8.4, *p* < 0.001; 37+ months: OR 8.1, 95% CI 3.6–18.1, *p* < 0.001), and the use of rain or bottled water (OR 5.6, 95% CI 1.3–23.8, *p* = 0.02; OR 1.9, 95% CI 1.0–3.4, *p* = 0.05) (Table 4).

Overall, there were 20 positive responses to questions about ‘Other activities’ that were highly likely to involve lead use or exposure, with only 6 of these children having BLLs >5 µg/dL. It is unknown whether any of these children represented duplicates from the same household. With regression analysis, there was no apparent increase in odds of having an elevated BLL with any of these specific variables or when combined into a group variable.

#### 3.5.2. Multivariate Analysis

Three variables were associated with relatively higher BLLs (>5 µg/dL) in the multivariate logistic regression model: province, water source, and a combined variable that included age and whether the child attended school outside the home. The other variables either made no significant additional contribution to the model, or there were too few children who had the risk factor and BLL >5 µg/dL (Table 5).

Two provinces, Binh Duong and Dong Nai, were associated with higher BLLs (OR 2.7, 95% CI 1.4–5.6, *p* = 0.01; OR 2.3, 95% CI 1.0–5.1, *p* = 0.05). The age and school variable did provide evidence that, as age increased, BLL increased. Excluding the 25–36-month-old children who did not attend school outside the home, the odds ratio for elevated BLL increased with age. The 25–36-month-old category did not show the same trend, but it was represented by a very small number of children (*n* = 7).

## 4. Discussion

Findings from this study support the premise that, although leaded gasoline has been phased out, there are still sources of lead exposure in Vietnam. However, it is now known that elevated BLLs are not ubiquitous throughout the country, as the prevalence of BLLs >10 µg/dL from this study was only 7.1%. This 7% is relevant, particularly considering the health effects caused by having elevated lead levels. For children with elevated levels, there may be acute symptoms of lead poisoning, which can be treated and hopefully resolved, or worse, there may be chronic effects with which they may need to cope throughout their entire life.

A similar prevalence of elevated BLLs and comparable overall mean BLLs has been seen in other countries throughout SE Asia. In Thailand, the predictors for higher BLLs have included peeling paint, eating paint chips, and geographic locationleading to average BLLs of 5.7 μg/dL—in 8.1% of the pediatric population studied by Chomchai et al. in 2005 [20]. In Malaysia, the primary predictor was living in an urban setting, and average BLLs were reported to be 5.3 μg/dL—a 6.4% prevalence [21]. In a study from the Philippines, it was noted that roof material, water source, hemoglobin, a history of breastfeeding, age, expenditure on cell phones, and home province were all associated with an increasing BLL (an average of 6.9 μg/dL with a prevalence of 21%) [22]. In Indonesia, Albalak et al. found that the level of education of the child’s primary caregiver, the water collection method, home varnishing, and living near a highway or major intersection all contributed to elevated BLLs that averaged 8.6 μg/dL and were seen in 35% of their tested population [23]. A study from China in 2011 indicated an average BLL of 6.2 μg/dL (8% of their participants) and found associations with being male, child’s age greater than 3, living in a crowded neighborhood, and smoking within the home [24].

**Figure 2:**
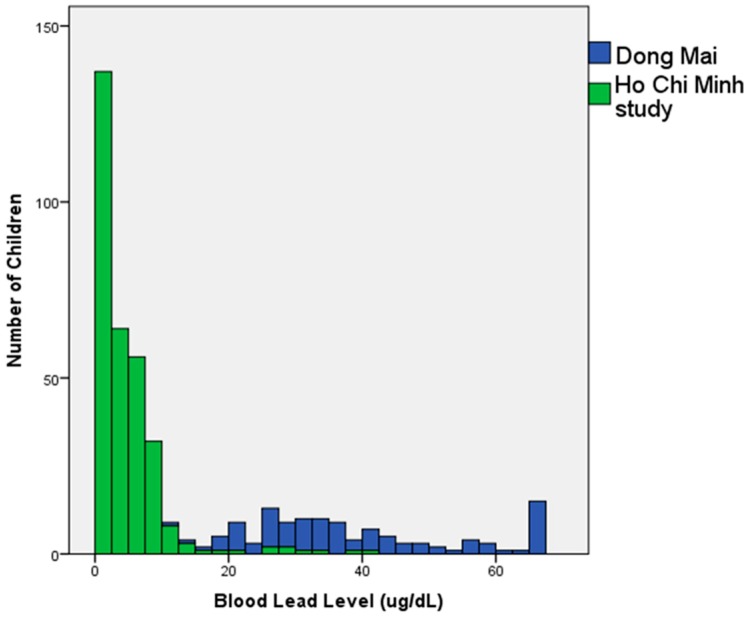
Comparison of child BLLs from the Dong Mai study (*n* = 109) [16] and Ho Chi Minh City (*n* = 311).

Living in or near a lead recycling village places children at risk. BLL levels measured in 109 children living in the lead recycling village of Dong Mai in early 2012 were quite elevated, clearly indicating that children in lead recycling villages warrant attention [16]. However, the children in this study, which largely represent populations residing in or near HCMC had markedly lower BLLs on average. This can be seen in Figure 2, which indicates the results of BLL testing from Dong Mai along with BLL results from HCMC. There was not a great deal of overlap in BLLs between these two populations. In the initial Dong Mai study, all children except for seven had a BLL >20 µg/dL, whereas in the HCMC study, only nine children had levels greater than 20 µg/dL [16].

In addition to proximity to lead recycling efforts, the risk factors for elevated BLL in Vietnam appear to include increasing age, drinking rainwater or possibly bottled water, and residing in Binh Duong or Dong Nai provinces. For screening purposes, these risk factors could be applied to the general pediatric population if it is assumed that the children in this study were a reasonable representation of the public.

It could be argued that this population of children, being ill and hospitalized, does not well represent the general population who might be exposed to lead. Researchers involved in the study thought that, if the children were hospitalized for respiratory concerns, these were not symptoms or diseases that tend to be associated with elevated BLLs and that, therefore, the children likely had no greater risk factors for lead exposure than the general population. If the children were often ill and treated with traditional medicines containing lead, this might increase their potential lead exposure, but this was not a risk for having elevated BLLs; conversely, if the children were frequently hospitalized, this could decrease the environmental lead exposure from their home or neighborhood. An additional concern is that of accuracy of the venipuncture testing process, used for confirmatory testing. We had to rely on the usual practices of the Vietnamese physicians, who obtained the services of a local laboratory. There were not enough paired samples (*n* = 3) (EAC lab and LeadCare II) to determine whether the testing methods correlated well.

There was a clear association between age and BLLs. The mean BLL was highest in 2-year-olds (6.71 µg/dL) compared to the other age groups, consistent with the concept that hand-to-mouthing activities are expected to put children of that age at higher risk [25]. The fact that the odds ratios for high BLL continued to increase with age does suggest that there may be ongoing lead exposures over time. Since the half-life of lead in bone is measured in years [26] and leaded gasoline was phased out in 2001, it remains a possibility that this effect may relate to the ongoing legacy of leaded gasoline exposure.

The significance of rainwater as a factor associated with elevated BLL is unclear. Information about income was not obtained as part of the research process, but rainwater use may be a surrogate marker for poverty or for living in a rural area. Another surrogate marker for poverty is education, but the highest educational level in the households that used rainwater was not lower than that of households that used other water sources. Additionally, educational levels in general did not have an association with elevated BLL. The information regarding water source was obtained during April and May, which is the dry season in Vietnam, and complicates the potential widespread nature of this variable. Explanations for elevated lead with rainwater use may include household storage containers that contain lead, collecting devices or roofing materials made with lead or lead solder, or reused industrial or plastic containers, particularly if there has been historical contamination of a site.

The etiology of possible lead exposures for children in Binh Duong and Dong Nai provinces is also not entirely clear. These two provinces are very near HCMC. Binh Duong has been considered “a favorable destination for mass infrastructure and industry,” [27] both of which may have an association with lead exposure. The lead battery industry has been present there, but no specific environmental reporting could be found to better describe potential sources for lead exposure.

Having such a broad assortment of variables associated with elevated BLLs in numerous countries in this study, yet so few that reached statistical significance, supports the need to have very community-specific evaluations by researchers who are culturally astute. Future questionnaires could be improved by gaining additional input from local community advisors to enhance any routine screening or surveillance process.

As Vietnam strives to become an economic powerhouse in SE Asia, the loss of ingenuity and productivity from 7% of their potential workforce is a significant cost that should not go unrecognized. After the phasing out of lead in gasoline in the USA, it was estimated that the cognitive capacity of American children would increase by 2–5 IQ points, leading to an estimated economic benefit of US $200 billion over the lifetime of each annual cohort born since 1980 [3]. On the other hand, universal pediatric blood lead testing is also a costly expenditure and likely not economically feasible. Therefore, targeted screening of at-risk populations remains the preferred approach. From a public health standpoint, knowing these sources is crucial in determining what is placing children at risk and how to avoid those exposures. This study did help identify possible at-risk groups, but increased characterization of potential sources of lead exposure is necessary.

This study was useful in bringing up the issue of lead exposure and initiating an effort to understand the extent of the problem. However, in addition to further lead testing in children, another aspect of alleviating this public health concern would be to provide education and training for the public health and medical personnel who may be caring for them. The goal of these efforts is to find ways to minimize lead exposure in children, ideally by understanding and modifying potential environmental exposures well enough that they can be completely avoided.

## 5. Conclusions

Our study increased characterization of lead exposures in a country that has yet to focus substantial attention on this issue. It is unfortunate that often the countries that are in the greatest need of surveillance may be those with the fewest resources to address the problems once they have been created. Improved surveillance of background lead levels can be a very beneficial initial stage in the process of creating relevant policies and focusing interventions for both in-country and global environmental remediation efforts. Although awareness is a critical first step in addressing lead-related health concerns, encouraging efforts to avoid or remediate environmental lead exposures, whether through public or private efforts, is ultimately the best approach to solving the lead problem.

## Figures and Tables

**Table 1:** Characteristics of participating children and their families, comparing children from Ho Chi Minh City and other provinces * (*n* = 311 **).

Child and Family Characteristics	HCMC	Other Provinces
Gender		
Male	91 (63%)	98 (59%)
Female	52 (36%)	67 (41%)
Age (months, mean and SD)	19.3 (22.0)	18.0 (19.0)
Height (cm, mean)	76.9 (17.1)	75.3 (14.6)
Weight (kg, mean)	10.2 (5.1)	9.8 (6.1)
Hemoglobin (g/dL, mean)	11.7 (1.5)	11.7 (2.6)
Hematocrit (%, mean)	36.1 (3.7)	35.6 (3.6)
Potential Lead Exposures		
Household member recycles	3 (2%)	2 (1%)
Child participates in recycling	0	1 (<1%)
Lead recycling in the house (in the past)	0	2 (1%)
Lead recycling in the home (currently)	0	2 (1%)
Using battery casings in the home for walls, furniture, or other purposes	0	1 (<1%)
Using battery casings outside the home for driveway, fences, or other purposes	0	1 (<1%)
Neighborhood lead recycling	2 (1%)	2 (1%)
Neighborhood burning of lead recycling waste	1 (<1%)	1 (<1%)
Metal recycling or melting	2 (1%)	4 (2%)
Occupational Exposures		
Parents do automotive work	4 (3%)	4 (2%)
Parents do ceramics/glazing	1 (<1%)	5 (3%)
Parents do welding	19 (13%)	31 (19%)
Environmental Exposures		
Source of Drinking Water		
Piped	65 (45%)	37 (22%)
Bottled	52 (36%)	43 (26%)
Rainwater	2 (1%)	7 (4%)
Well Water	26 (18%)	78 (47%)
Peeling paint	48 (34%)	47 (29%)
Live near a busy intersection	27 (19%)	58 (35%)
Use traditional medicines	117 (81%)	108 (65%)
Age of home (mean) (years)	10.8 (10.3)	8.67 (6.7)
Home is painted-outside	115 (80%)	125 (76%)
Home is painted-inside	124 (86%)	131 (79%)
Years since home was most recently painted	3.65 (4.3)	3.9 (4.0)
Symptoms		
Abdominal Pain	10 (7%)	12 (7%)
Constipation	12 (8%)	17 (10%)
Educational Factors		
Highest level of education in the home		
None	2 (1%)	2 (1%)
Grade School	47 (33%)	69 (42%)
High School	53 (37%)	71 (43%)
University Degree	40 (28%)	24 (14%)
University Degree +***	2 (1%)	0
Child has problems learning in school	5 (3%)	9 (5%)
Attends school outside the home	35 (24%)	42 (25%)

* Table displays counts (and percent of column total) or means (and standard deviations). ** Numerical discrepancies are due to missing information. *** University Degree + = more than 4 years of undergraduate studies.

**Table 2:** Mean and median (interquartile range (IQR)) BLLs by province.

Province	Number (% of Total)	Mean BLL (μg/dL)	Median BLL (μg/dL) IQR
Binh Duong	59 (19%)	6.0	5.4 (1.65–7.9)
Binh Phuoc	21 (6.8%)	4.2	1.7 (1.65–3.8)
Dong Nai	39 (12.5%)	6.7	4.0 (1.65–7.7)
Dak Nong	5 (1.6%)	4.9	3.8 (1.65–4.1)
HCM	145 (46.6%)	4.5	1.7 (1.65–5.4)
Lam Dong	8 (2.6%)	3.3	3.6 (1.65–4.2)
Ba Ria-Vung Tau	13 (4.2%)	2.7	1.7 (1.65–3.5)
Other *	21 (6.8%)	5.3	4.8 (1.65–7.4)

* Other includes: Binh Thuan (3), Dak Nong (4), Kien Giang (2), Long An (2), Phu Yen (2), Ben Tre (1), Dak Lak (1), Kon Tum (1), Lang Son (1), Quang Nam (1), Thanh Hoa (1), Tien Giang (1), Vinh Long (1).

**Table 3:** BLLs relative to major child and family characteristics (*n* = 311).

Child or Family Characteristic	Mean (µg/dL)	Median (µg/dL) and IQI	*p*-Value *
Gender			0.77
Male	5.19	3.70 (1.65–6.52)	
Female	4.63	3.60 (1.65–5.80)	
Age (months)			<0.001
0–12	3.75	1.65 (1.65–3.60)	
13–24	6.71	5.20 (3.63–7.60)	
25–36	5.39	4.90 (1.65–7.40)	
37+	5.70	5.50 (3.70–7.90)	
Location			0.02
Ho Chi Minh	4.41	1.65 (1.65–5.35)	
All other locations	5.43	3.80 (1.65–6.70)	
Water source			0.12
Piped	4.54	1.65 (1.65–5.20)	
Bottled	4.88	4.00 (1.65–6.20)	
Rainwater	5.62	5.40 (3.28–7.95)	
Well water	5.39	3.65 (1.65–6.68)	
Use traditional medicines	5.12	3.80 (1.65–6.20)	0.43
Have peeling paint in the home	4.84	3.50 (1.65–5.70)	0.61
Attends school outside the home	6.54	5.70 (4.10–8.15)	<0.001
Parents do metal recycling	10.64	5.45 (4.39–14.55)	0.12
Live near a busy intersection	5.14	3.60 (1.65–6.35)	0.43

* Significance (*p*-value) based on Mann–Whitney U or Kruskal–Wallis tests.

**Table 4:** Risk of high BLLs (>5 µg/dL, *n* = 109), relative to child and family characteristics, by bivariate analysis.

Variable	Number and % of Children	Odds Ratio and 95% Confidence Interval	*p*-Value
Gender			
Male	67 (61%)	1 *	
Female	42 (39%)	0.99 (0.6–1.6)	0.99
Age (months)			
0–12	29 (27%)	1	
13–24	38 (35%)	4.4 (2.4–8.1))	<0.001
25–36	19 (17%)	4.0 (1.9–8.4)	<0.001
37+	23 (21%)	8.1 (3.6–18.1)	<0.001
School outside the home	48 (44%)	0.2 (0.1–0.4)	<0.001
Busy intersection near home	27 (25%)	1.3 (0.7–2.1)	0.40
Use of traditional medicine	46 (42%)	0.9 (0.5–1.4)	0.54
Welding	21 (19%)	0.8 (0.4–1.4)	0.43
Metal recycling or melting	5 (5%)	0.1 (0.0–0.9)	0.04
Water Source			
Piped	27 (25%)	1	
Bottled	38 (35%)	1.9 (1.0–3.4)	0.05
Rainwater	6 (6%)	5.6 (1.3–23.8)	0.02
Well	38 (35%)	1.6 (0.9–2.9)	0.12
Other activities **	6 (6%)	**	

* A variable category with odds ratio = 1 is the reference category for that variable. ** Other activities: household member recycles, child participates in recycling, lead recycling occurs in the home, either currently or in the past, battery casings are used in or around the home, neighborhood lead recycling is present, or neighborhood burning of lead recycling waste is occurring.

**Table 5:** Odds of higher BLLs (>5 μg/dL, *n* = 109) by multivariate analysis.

Variable	Number (%)	OR (95% CI)	*p*-Value
Province			
Ho Chi Minh	41 (38%)	1 *	
Binh Duong	33 (30%)	2.7 (1.4–5.6)	0.01
Binh Phuoc	4 (4%)	0.5 (0.1–1.8)	0.30
Dong Nai	18 (17%)	2.3 (1.0–5.1)	0.05
Lam Dong	1 (1%)	0.2 (0.0–2.3)	0.20
Ba Ria	3 (3%)	0.5 (0.1–2.5)	0.39
Other	8 (7%)	2.1 (0.7–6.2)	0.32
Water Source			
Piped	27 (25%)	1	
Bottled	38 (35%)	1.8 (0.9–3.3)	0.08
Rainwater	6 (6%)	3.6 (0.8–16.23)	0.09
Well Water	38 (35%)	1.5 (0.8–2.9)	0.23
Age & School			
0–12 months	24 (22%)	1	
13–24 months	42 (39%)	6.0 (3.1–11.8)	<0.001
25–36 months			
Attends school	17 (16%)	7.5 (2.8–19.9)	<0.001
Not in school	2 (2%)	0.9 (0.2–5.0)	0.90
>37 months			
Attends school	17 (16%)	12.6 (4.6–34.3)	<0.001
Not in school	7 (6%)	16.8 (3.8–74.7)	<0.001

* A variable category with odds ratio = 1 is the reference category for that variable.
